# Cognitive, adaptive and daily life functioning in adults with 22q11.2 deletion syndrome

**DOI:** 10.1192/bjo.2024.745

**Published:** 2024-11-11

**Authors:** Claudia Vingerhoets, Julia Ruiz-Fernandez, Emma von Scheibler, Elfi Vergaelen, Nele Volbragt, Nele Soons, Chaira Serrarens, Annick Vogels, Erik Boot, Therese van Amelsvoort, Ann Swillen

**Affiliations:** Department of Psychiatry and Neuropsychology, MHeNs, Maastricht University, Maastricht, The Netherlands; and Advisium, ‘s Heeren Loo Zorggroep, Amersfoort, The Netherlands; Department of Psychiatry and Neuropsychology, MHeNs, Maastricht University, Maastricht, The Netherlands; and INSERM U1299, Centre Borelli UMR9010, ENS-Paris-Saclay, Université Paris Saclay, Paris, France; Department of Psychiatry and Neuropsychology, MHeNs, Maastricht University, Maastricht, The Netherlands; and Koraal, Maastricht, The Netherlands; Center for Human Genetics, University Hospital Leuven, KU Leuven, Leuven, Belgium; and Department of Human Genetics, University Hospital Leuven, KU Leuven, Leuven, Belgium; Department of Psychiatry and Neuropsychology, MHeNs, Maastricht University, Maastricht, The Netherlands; Department of Psychiatry and Neuropsychology, MHeNs, Maastricht University, Maastricht, The Netherlands; Advisium, ‘s Heeren Loo Zorggroep, Amersfoort, The Netherlands; and The Dalglish Family 22q Clinic, Toronto, Ontario, Canada

**Keywords:** 22q11.2DS, cognition, adaptive functioning, daily life functioning, adults

## Abstract

**Background:**

22q11.2 deletion syndrome (22q11.2DS) is associated with cognitive impairments and an increased risk of psychopathology. Most of the research has been conducted in children and adolescents, although the majority of affected individuals live well into adulthood. Hence, limited data are available on functional outcomes in adults.

**Aims:**

To provide more insight in cognitive and adaptive abilities, and daily life functioning (marital status, living situation and work situation) in adults with 22q11.2DS.

**Method:**

This retrospective study included 250 Dutch-speaking adults (16–69 years) with 22q11.2DS from three sites in The Netherlands and Belgium. Data on full-scale IQ (FSIQ) scores (assessed with the Wechsler Adult Intelligence Scale), adaptive functioning (assessed with the Vineland Adaptive Behavior Scale II), and functional outcomes including marital status, living and work situation were systematically collected from clinical files. In addition, we examined predictors of adaptive functioning.

**Results:**

The majority of individuals in our adult sample demonstrated a low level of adaptive functioning (65%). In contrast to previous findings in children and adolescents, the majority functioned at an intellectual disability level (56%). Male sex, lower FSIQ and autism spectrum disorder were predictors of lower adaptive functioning (*P* = 0.016, *P* < 0.001 and *P* = 0.16, respectively).

**Conclusions:**

These results suggest that low levels of cognitive and adaptive functioning are common in adults with 22q11.2DS. Future longitudinal and multicentre studies including older patients (>40 years) are needed to further investigate cognitive and adaptive trajectories and their interactions with physical and psychiatric comorbidities.

22q11.2 deletion syndrome (22q11.2DS) is a multisystem disorder caused by a microdeletion on chromosome 22. With an estimated prevalence of one in 2148 births,^1^ it is among the most common microdeletion syndromes in humans. The phenotype is highly variable and includes congenital heart disease, palatal abnormalities, immunodeficiency and characteristic facial features.^2,3^ Moreover, psychiatric disorders are common across the lifespan. In children and adolescents, a high prevalence of neurodevelopmental disorders, including attention-deficit hyperactivity disorder (ADHD, up to ~40%), autism spectrum disorders (ASD, up to ~30%) and anxiety disorders (up to ~35%), has been reported.^4^ At adult age, anxiety disorders remain common, with approximately 2–3 times the expected population prevalence, as well as psychotic disorders, with a 20-fold increased risk compared with the general population.^2^ Regarding overall development, learning problems, cognitive deficits and intellectual disability are often present.^2,4^ In children, verbal IQ (VIQ) often exceeds performance IQ (PIQ) by >10 points.^4^ A modest but significant decline, most prominent in VIQ, has been described in a subgroup of children and adolescents with 22q11.2DS throughout development, particularly in individuals who developed a psychotic disorder.^5^ Moreover, cognitive decline has been reported in a sample of adults with 22q11.2DS,^6^ suggesting that cognitive abilities may not be stable over time.^7^ Cognitive decline has been found to be steeper in those individuals developing psychosis.^5^

Most of the studies in 22q11.2DS to date have been performed in children and adolescents, although most individuals live well into adulthood.^8^ Therefore, less is known about cognitive, adaptive and daily life functioning in adulthood. Very few studies on cognitive functioning in adults with 22q11.2DS are available.^9,10^ These studies reported impairments in visual–perceptual abilities, problem-solving and planning, and abstract and social reasoning^10^ and found that cognitive functions generally were more impaired in patients with comorbid schizophrenia.^9^

In addition to cognitive impairments, low levels of adaptive functioning have been reported in adults with 22q11.2DS.^8,11^ Adaptive functioning refers to the set of personal and social skills necessary to navigate through daily life and cope with environmental demands.^12^ In a previous study by Butcher et al,^8^ 75% of individuals with 22q11.2DS (*N* = 100, mean age of 29 years) scored in the functional deficit range, with daily living skills as a relative strength. Adaptive functioning was predicted by full-scale IQ (FSIQ) and having a diagnosis of schizophrenia. In their sample, 9% had an IQ in the average range, 48% had borderline intelligence and 43% had a mild intellectual disability. However, Butcher et al excluded patients with a moderate or severe intellectual disability to prevent possible floor effects. Therefore, given the absence of other studies in adults, the proportion of adults with 22q11DS functioning at a moderate or severe intellectual disability level remains unknown. Nonetheless, studies conducted in children and adolescents have reported that borderline intelligence and mild intellectual disability are common, whereas average intellectual functioning and moderate to severe intellectual disability are less often present.^4,13,14^

Information about cognitive, adaptive and daily life functioning at adult age is important to counsel patients, families, and clinicians and to provide targeted remediation, interventions and support for patients and their families. For example, levels of adaptive functioning were higher in employed adults with 22q11DS compared with those who were unemployed.^15^ Moreover, adaptive functioning more than cognitive functioning was found to predict employment status in adults with 22q11DS.^16^ In addition, married individuals with 22q11DS showed higher adaptive functioning and fewer psychotic comorbidities compared with unmarried individuals with 22q11DS.^17^ Therefore, the main aim of the present study was to gain more insight into the cognitive abilities, adaptive functioning and functional outcomes (marital status, living situation and work situation) of a large group of adults with 22q11.2DS. The secondary aim was to identify predictors of adaptive functioning. As cognitive decline and low adaptive functioning have been repeatedly described in a subgroup of patients, we hypothesised that on average, adults with 22q11.2DS would function at a lower intellectual and adaptive level than previously described in children with 22q11.2DS.

## Method

The authors assert that all procedures contributing to this work comply with the ethical standards of the relevant national and institutional committees on human experimentation and with the Helsinki Declaration of 1975, as revised in 2008. This was a retrospective study based on data derived from scientific research as well as clinical files. All research procedures involving human subjects were approved by the Medical Ethics Committee of the Maastricht University Medical Centre, Maastricht, The Netherlands (NL70681.068.19) and the Ethical Committee of UZ Leuven (S 52418). Ethical approval for the use of clinical case files was waived by the respective local ethical committees. Nevertheless, all patients and/or their legal representatives provided written informed consent for their data to be used for scientific research. All data were processed anonymously.

### Participants

For this study, case files were collected for 250 patients who had visited the specialised 22q11 copy number variant out-patient clinic of Maastricht University Medical Center+, Maastricht, The Netherlands (*N* = 127); ‘s Heeren Loo (a facility for people with an intellectual disability), Amersfoort, The Netherlands (*N* = 57); and the Center for Human Genetics at the University Hospital Gasthuisberg, Leuven, Belgium (*N* = 66) were collected. All subjects had a formal diagnosis of 22q11.2 microdeletion based on fluorescence *in situ* hybridisation or microarray. Generally, individuals with 22q11DS are referred to these specialised clinics by their general practitioner, a medical specialist (e.g. cardiologist, clinical geneticist, intellectual disability physician) or a specialised clinic for children with 22q11DS. Reasons for referral are typically consultation on medical and/or psychiatric problems or 22q11DS-oriented follow-up as advised in the clinical guidelines.^2^ Individuals can also be referred at their own request by their general practitioner. All participants were aged between 16 and 69 years with a mean age of 28.66 years (s.d. 11.95) at the time of the most recent clinical or research assessment. Assessments conducted before the age of 16 were not taken into account. For some participants, measurement of IQ or adaptive functioning had been conducted before the most recent clinical assessment but always at an age ≥16 years (e.g. most recent clinical assessment at the age of 24 but most recent IQ measurements at the age of 20 years). Supplementary Table 1 available at https://doi.org/10.1192/bjo.2024.745 displays the mean ages for the separate assessments. A 22q11.2 microdeletion was confirmed for all participants by either multiplex ligation-dependent probe amplification analysis, fluorescence *in situ* hybridisation with a standard 22q11.2 region probe, or microarray (comparative genomic hybridisation).

### Assessments

#### IQ

IQ scores were assessed with an age-appropriate version of the Wechsler Intelligence Scale. A large number of individuals (*N* = 116) were assessed with a shortened version of the Wechsler Adult Intelligence Scale III.^18^ Therefore, information about working memory and processing speed was not available for many participants. For an overview of the tests used for IQ assessment, see Supplementary Table 1.

### Adaptive functioning

Adaptive functioning was assessed with the Vineland Adaptive Behaviour Scale (VABS II).^12^ The VABS is a semi-structured interview assessing three core domains of adaptive functioning: communication, daily living skills (practical skills necessary in daily life such as self-care and domestic tasks) and socialisation (skills necessary to interact with others, regulate emotions, and engage in social and leisure activities). From these three domains, an overall adaptive behavioural score can be computed, with higher scores indicating a higher level of adaptive functioning. Standardised scores were reported, as well as developmental age equivalents. For some patients, only developmental age equivalents were available from the clinical file. In these cases, standardised scores were calculated from the tables provided in the manual.^12^ When the age equivalent corresponded to a range of standardised scores, the highest score of that range was used.

### Level of intellectual disability

The presence or absence of intellectual disability, and its severity, were determined based on all information available on intellectual functioning and adaptive functioning in daily life, in addition to FSIQ scores, and according to the DSM-5. This means that not only FSIQ but also adaptive functioning was taken into account in determining (the level of) intellectual disability. For example, an individual with a FSIQ of 74 and significant impairments in daily living skills (e.g. in need of daily assistance with self-hygiene and meal preparation) would be classified as having mild intellectual disability. An overview of FSIQ and level of intellectual disability is provided in Supplementary Table 2. For the majority of participants, the FSIQ corresponded to the clinical assessment based on the DSM-5.

### Functional outcomes

Living situation, work and marital status were assessed as markers of functional outcome. These variables were consistently reported in all clinical case files. These variables were also assessed in a standardised manner for scientific research and therefore chosen as indicators for functional outcome. Marital and employment status have previously been found to be predicted by adaptive functioning in 22q11DS.

### Procedures

All data were gathered in the context of scientific research and/or clinical care. Clinical case files were screened for information about FSIQ scores, adaptive functioning, psychopathology and functional outcomes (marital status, living situation and work). All assessments were conducted by a trained and experienced research assistant or experienced clinician. Psychiatric diagnoses (according to the DSM-5) were gathered from the clinical case files. For the individuals that participated in scientific research, the GOASSESS^19^ interview was administered by an experienced research assistant and/or an experienced psychiatrist. The GOASSESS covers a wide variety of psychiatric symptomatology including mood and anxiety.^19^ Based on this information and additional clinical information, an experienced psychiatrist determined the diagnosis. In cases where both research data and clinical data were available, we used the most recent research data because of the standardised method of assessment. Hence, subjects seen at two or more sites were assigned to the site where research data were obtained.

### Statistical analyses

All statistical analyses were performed in SPSS version 28 (IBM SPSS Statistics). Demographics were compared between the three sites using chi-squared test (sex), Kruskal–Wallis test (age) and univariate analysis of variance (FSIQ, VIQ and PIQ). In addition, we computed the prevalence of intellectual disability and adaptive functioning deficits, psychopathology and several functional outcome variables (e.g. marital and vocational status). Multivariate linear regression models were used to identify predictors of overall adaptive functioning, as well as separate subdomains considering age, sex, FSIQ and history of psychopathology (diagnoses) as predictor variables. All four models were checked for multicollinearity. We also compared intellectual functioning and presence of psychopathology between male and female adults using chi-squared analyses. Sex differences in adaptive functioning were examined using multivariate analyses of variance. Finally, Pearson correlation coefficients were computed to explore possible associations between age and adaptive functioning. The main analyses were repeated in a group excluding subjects below the age of 18.

## Results

Sample demographics and IQ scores for the total sample as well as samples per site are displayed in Table 1. The total sample consisted of 250 adults with 22q11.2DS. Supplementary Table 1 displays the mean age per assessment (IQ, adaptive functioning and functional outcomes).

**Table 1:** Sample demographics and IQ scores

	Total sample *N* = 250 Mean (s.d.)	Maastricht *n* = 127 Mean (s.d.)	‘s Heeren Loo *n* = 57 Mean (s.d.)	Leuven *n* = 66 Mean (s.d.)	*P*
Sex, male/female	109/141	52/75	26/31	31/35	0.683
Age, years^a^	28.7 (12.0)	31.0 (10.5)	32.4 (15.2)	20.83 (7.0)	<0.001
FSIQ^b^	70.5 (11.6)	72.7 (11.0)	62.6 (10.7)	70.9 (11.6)	<0.001
VIQ	71.6 (12.8)	72.2 (13.3)	70.8 (12.5)	70.8 (12.3)	0.855
PIQ	69.5 (13.8)	68.7 (14.8)	66.9 (11.1)	72.2 (13.0)	0.073
*De novo*/familial/unknown	146/22/82	73/11/43	19/1/37	54/10/2	0.470

FSIQ, full-scale IQ; VIQ, verbal IQ; PIQ, performance IQ.

a. Mean age was significantly lower in the Leuven sample compared with the Maastricht and ‘s Heeren Loo samples (*P* < 0.001).

b. Mean FSIQ scores were significantly lower in the ‘s Heeren Loo sample compared with the Maastricht and Leuven samples (*P* < 0.001 and *P* = 0.001, respectively).

### Intellectual level of functioning

Only 7% of adults with 22q11.2DS had intelligence scores within the average range (FSIQ 85–115). Approximately 36% had borderline intelligence. Fifty-six per cent functioned at the level of intellectual disability, of which the majority met criteria for a mild intellectual disability (43.4%). A moderate or severe intellectual disability level of functioning was present in approximately 13% of the patients. The results are displayed in Table 2.

**Table 2:** Level of intellectual and adaptive functioning in adults with 22q11.2DS

Level of intellectual functioning^a^	*N* (total 250)	Percentage
Average intelligence	17	6.8
Borderline intelligence	91	36.4
Mild intellectual disability	108	43.2
Moderate intellectual disability	25	10.0
Severe/profound intellectual disability	9	3.2

Level of adaptive functioning^b^	*N* (total 163)^a^	Percentage
High	0	0.0
Moderately high	1	0.6
Adequate	28	17.2
Moderately low	28	17.2
Low	106	65.0

Adaptive functioning domain scores	*N*	Standardised score	Age equivalent
Communication	162	53.1	9 years and 2 months
Daily living skills	162	69.1	11 years and 4 months
Socialisation	162	60.4	9 years and 8 months
Overall adaptive functioning	163	58.0	

a. Level of intellectual disability based on DSM-5 criteria or only full-scale IQ (FSIQ) if no additional data were available. Average intelligence: FSIQ 85–115; borderline intelligence, FSIQ 70–84; mild intellectual disability, FSIQ 50–69; moderate intellectual disability: FSIQ 35–49; severe/profound intellectual disability FSIQ <35.

b. Vineland Adaptive Behaviour Scale scores were available for 163 of the 250 adults. Adaptive functioning classifications were based on standard scores: high: 131–160; moderately high: 116–130; adequate: 85–115; moderately low: 70–84; low: 20–69.

### Adaptive functioning

The mean age at time of last assessment was 30.3 years (s.d. 10.26). The majority of adults had low levels of adaptive functioning, with developmental age equivalent ranging from 9 years and 2 months for the communication domain, 9 years and 8 months for the socialisation domain, and 11 years and 4 months for daily living skills. The results are displayed in Table 2.

Multivariate linear regression analyses yielded significant predictive models for overall adaptive functioning (*P* < 0.001, adjusted *R*^2^ = 0.395), communication (*P* < 0.001, adjusted *R*^2^ = 0.380), daily living skills (*P* < 0.001, adjusted *R*^2^ = 0.346) and socialisation (*P* < 0.001, adjusted *R*^2^ = 0.246). All models were checked for multicollinearity using variance inflation factor and tolerance scores. Variance inflation scores were all below 5 (range 1.048–1.270), and tolerance scores were below 0.1 (0.786–0.954). In addition, Pearson's correlation statistics for predictor variables were all <0.70, with a maximum value of 0.395 for the correlation between age and depression. FSIQ and ASD significantly predicted overall adaptive functioning, as well as communication, daily living skills and socialisation (Table 3). Sex predicted overall adaptive functioning, daily living skills and socialisation but not communication (Table 3). Age at assessment only predicted daily living skills. Finally, having an anxiety disorder predicted communication. Notably, depression, psychosis and ADHD did not significantly predict adaptive functioning. Regression models including VIQ and PIQ did not predict adaptive functioning in an additional explorative model (data not presented).

**Table 3:** Predictors of adaptive functioning^a^

	Overall adaptive functioning		Communication		Daily living skills		Socialisation	
	*B*	*P*	*B*	*P*	*B*	*P*	*B*	*P*
Age	0.34	0.058	0.16	0.456	0.57	**0.004**	0.37	0.067
Sex	11.90	**0.005**	9.56	0.063	12.13	**0.011**	11.59	**0.016**
FSIQ	0.84	**<0.001**	1.04	**<0.001**	0.84	**<0.001**	0.73	**<0.001**
Depression	1.14	0.802	7.97	0.153	−1.55	0.760	−0.92	0.858
Psychosis	−1.95	0.717	−1.78	0.786	−3.66	0.542	2.28	0.708
Anxiety	−9.84	0.111	−17.95	**0.018**	−1.79	0.794	−5.59	0.422
ADHD	7.05	0.457	4.56	0.692	5.67	0.590	13.42	0.211
ASD	−14.37	**0.005**	−16.08	**0.012**	−11.38	**0.050**	−12.59	**0.016**

FSIQ, full-scale IQ; ADHD, attention-deficit hyperactivity disorder; ASD, autism spectrum disorder.

a. Numbers in bold reflect significant predictors.

### Functional outcomes

Functional outcomes are presented in Table 4.

**Table 4:** Functional outcomes in adults with 22q11.2DS

Functional outcomes	*N* (total 246)^a^	Percentage
Marital status		
Single (never married)	188/246	76.4
Married	33/246	13.4
Long-term relationship	16/246	6.5
Divorced	5/246	2.0
Widowed	4/246	1.6
Living situation		
With parents/family	92/246	37.4
With spouse/own family	52/246	21.1
intellectual disability setting	50/246	20.3
Independent (alone)	27/246	11.0
Protected environment (alone)	12/246	4.9
Psychiatric setting	9/246	3.7
With roommates	2/246	0.8
Other	2/246	0.8
Work/day care		
Day centrer	63/243	26.0
Sheltered work	58/243	23.9
Regular job part-time	40/243	16.5
Unemployed/no daytime activities	25/243	10.3
School	23/243	9.5
Regular job full-time	19/243	7.8
Household	8/243	3.3
Volunteer work	7/243	2.9

a. Information on functional outcome was available for 246 of the 250 adults.

### Psychopathology

At least one psychiatric or neurodevelopmental disorder was present in 52% of the individuals (*n* = 244). Within the total sample, 31% had one psychiatric diagnosis, 17% had two diagnoses, 3% had three diagnoses and 1% had four diagnoses. ASD (22%) and psychotic disorders (20%) were most often observed, followed by depression (19%) and anxiety disorders (16%). ADHD (7.1%) and bipolar disorder (0.8%) were the least prevalent in our sample. The results are reported in Supplementary Table 3.

### Sex differences

No significant differences were found in FSIQ (*t*(226) = −1.04, *P* = 0.300), VIQ (*t*(214) = −1.13, *P* = 0.262) or PIQ (*t*(215) = −0.99, *P* = 0.325) between male and female adults. However, males were 1.7 times more likely to have an intellectual disability than females (odds ratio 1.7; 95% CI 1.01–2.83; *P* = 0.044). Women were significantly more likely to function at a borderline intelligence level than men (odds ratio 1.8; 95% CI 1.07–3.13; *P* = 0.026). No sex differences were found between average intelligence, mild intellectual disability, moderate intellectual disability or severe intellectual disability levels of functioning (Supplementary Table 4).

Adaptive functioning was compared between males (*N* = 67) and females (*N* = 96) (Supplementary Table 5). The results demonstrated higher overall adaptive functioning (*F*(4, 157) = 6.21, *P* = 0.014, η^2^ = 0.37) and daily living skills (*F*(4, 157) = 7.32, *P* = 0.008, η^2^ = 0.44) in females compared with males.

There was no statistically significant difference in the proportion of adults with history of a psychiatric disorder between males (59%) and females (47%). However, males were 2.4 times more likely to have a psychotic disorder (odds ratio 2.4; 95% CI 1.28–4.51; *P* = 0.006) and 2.2 times more likely to have ASD (odds ratio 2.2; 95% CI 1.16–4.24; *P* = 0.016) (Supplementary Table 6). Functional outcomes for males and females separately are presented in Supplementary Table 7.

### Age effects

**Fig. 1:**
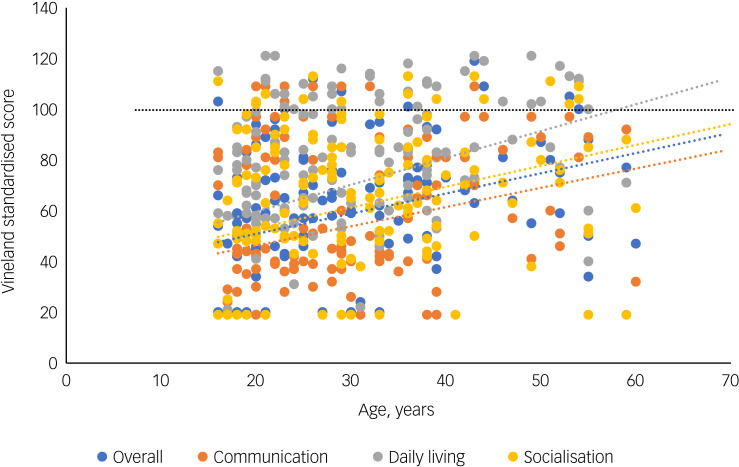
Relationships of age with overall adaptive functioning (Vineland Adaptive Behaviour Scale), communication, daily living skills and socialisation. The black dotted line represents the mean standardised score within the norm population (average adaptive functioning).

No significant associations were found between age and FSIQ (*r* = −0.011, *P* = 0.869), VIQ (*r* = 0.009, *P* = 0.892) or PIQ (*r* = 0.018, *P* = 0.793). As psychosis is associated with cognitive decline, we examined the relationship between age and FSIQ (*r* = −0.285, *P* = 0.079), VIQ (*r* = −0.113, *P* = 0.524) and PIQ (*r* = −0.003, *P* = 0.988) in a subgroup of adults diagnosed with a psychotic disorder, but again we found no significant associations. Age at time of assessment was significantly positively associated with overall adaptive functioning (*r* = 0.224, *P* = 0.004) as well as with the communication (*r* = 0.206, *P* = 0.009), daily living (*r* = 0.236, *P* = 0.003) and socialisation (*r* = 0.219, *P* = 0.005) subscales (Fig. 1). Adaptive functioning was higher with increasing age. The main analyses were repeated excluding individuals ≤18 years old; the results remained comparable (Supplementary Tables 8–10).

## Discussion

The present study examined cognitive, adaptive and daily life functioning in a large group of adults with 22q11.2DS. Overall, we report a relatively high percentage of adults functioning at an intellectual disability level, with the majority meeting criteria for mild intellectual disability, and low levels of adaptive functioning, particularly in the domains of communication and socialisation.

In contrast to the findings of studies on intelligence and cognitive functioning in children and adolescents,^4,7,13^ the majority of individuals in our sample met DSM-5 criteria for an intellectual disability. The number (56%) was also higher than that reported by a previous study in adults.^8^ However, that study did not include adults with 22q11.2DS with moderate and severe intellectual disabilities. In the current sample, we found that approximately 13% of adults met criteria for moderate and severe intellectual disability, suggesting that these levels of (intellectual and adaptive) functioning are more common in adults with 22q11.2DS than previously assumed. A recent study by Leader et al^11^ reported an intellectual disability prevalence of 85%, with moderate and severe intellectual disability present in 40% of 101 adults with 22q11.2DS recruited through 22q11 patient and family support groups. However, these numbers were based on proxy reports (parents). In our study, levels of intellectual disability were based on DSM-5 criteria assessed by formal IQ and adaptive behaviour assessments and/or by experienced clinicians.

There are several possible explanations for the relatively high proportion of adults functioning at intellectual disability level compared with previous research in children. First, these results could be partly explained by ascertainment bias, as a subgroup of adults was recruited through an intellectual disability facility. Moreover, cognitive decline has been described in a subgroup of children and young adults with 22q11.2DS. Such decline may be related to the development of mental health problems.^6^ In addition, adults with 22q11.2DS are at increased risk of developing Parkinson's disease,^2^ a neurodegenerative disorder associated with cognitive deficits and dementia, from a relatively young age. The mean age of our adult sample was relatively young. Therefore, future studies including older patients should further investigate a potential premature ageing effect. Another possible explanation for the relatively high percentage of adults functioning at an intellectual disability level may represent a ‘growing into deficits trajectory’, meaning that insufficient cognitive development leads to increased discrepancy relative to age-required norms.^20^ Notably, we did not find an association between age and FSIQ in our adult sample, which may suggest that the ‘growing into deficits trajectory’ is more likely than a degenerative cognitive decline (in terms of a decline in ‘raw IQ scores’).

Another explanation for the discrepancy in findings could be that we used DSM 5 criteria to determine the level of intellectual disability, in contrast to previous studies. This allows for a more comprehensive view of the individual's capacities for daily life functioning, as more emphasis is put on adaptive behaviour, and generates a better representation of daily life functioning than use of IQ scores alone. Given the large number of adults in our sample with adaptive skills level in the moderately low and low range, this could explain the relative high prevalence of intellectual disability. Moreover, it may explain why intellectual disability was more often present in male patients than in females, as males presented with lower levels of adaptive functioning, whereas no sex differences were found in terms of IQ. However, in the general population, intellectual disability is more common in males than females. Therefore, this finding may not be characteristic of individuals with 22q11.2DS.^21^

### Adaptive functioning

In line with Butcher et al,^8^ we found that the majority of adults with 22q11.2DS scored within the functional deficits range on the VABS. Overall, adaptive functioning was predicted by FSIQ, sex and having a diagnosis of ASD. Contrary to previous findings,^8^ history of a psychotic disorder did not predict adaptive functioning in our sample. As expected, patients with higher FSIQ demonstrated higher levels of overall adaptive functioning, as well as higher functioning on the separate domains of communication, daily living skills and socialisation. Increasing age was associated with a higher level of daily living skills, suggesting that adults with 22q11.2DS are able to acquire these skills, although this may occur at a slower pace. Importantly, this suggests that adaptive functioning may not decline with increasing age. However, this was a cross-sectional study, and our sample of adults was relatively young. Therefore, longitudinal studies are necessary to further investigate this. Our findings also imply that adults with 22q11.2DS with a diagnosis of ASD have lower adaptive functioning across all domains. This is in line with findings of lower adaptive functioning in children and adolescents with idiopathic ASD.^22^ Finally, females with 22q11.2DS had higher levels of overall adaptive functioning and daily living skills compared with males. The presence of ASD may mediate this relationship.

### Psychopathology

In line with previous studies in adults with 22q11.2DS, we report high rates of psychopathology.^11,23,24^ In our sample, approximately half of the adults had been diagnosed with at least one psychiatric disorder. Additional psychiatric comorbidities were relatively common. In line with previous findings, the most common diagnoses were psychosis spectrum disorders and ASD.^2^ In contrast to previous studies of 22q11.2DS, the prevalence of anxiety disorders and ADHD was relatively low in our sample.^23,24^ Our sample included more adults with a moderate or severe intellectual disability compared with previous studies, which may have caused diagnostic overshadowing and underdiagnosis of anxiety disorders and ADHD. However, previous research has demonstrated a lower prevalence of ADHD in adults with 22q11DS (~16%) compared with children (~37%) and adolescents (~24%),^23^ suggesting that these symptoms do not always persist in adulthood. Nevertheless, the low prevalence of ADHD may have been caused by diagnostic overshadowing. Indeed, an earlier study reported that ADHD was overshadowed in 71.8% of cases with neurological learning disabilities.^25^ Male participants with 22q11.2DS had increased risk of ASD and psychotic disorders compared with females. This is comparable with the general population, in which males are more often diagnosed with psychosis and ASD than females.^26,27^

### Functional outcomes

Our results showed that only a limited number of adults with 22q11.2DS had a regular (paid) full-time job. Most employed participants worked part-time or had an adjusted job in a protected environment. This is in contrast to the findings of Mosheva et al,^16^ who reported that 33.3% of their sample of 138 adults with 22q11 were in open market employment and 25.4% in assisted employment, and 41.3% were unemployed. Moreover, Curtin et al reported that 24.8% of their sample of 101 adults with 22q11DS were employed.^15^ This may be related to the low levels of adaptive functioning, as these skills are necessary to get and maintain a job. Indeed, Mosheva et al^16^ found that employment was predicted by adaptive functioning. In our sample, the level of adaptive functioning was generally low. In addition, physical problems, which were frequently present, may have limited the ability of participants to maintain employment. Moreover, deficits in executive functioning are often observed in children and (young) adults with 22q11.2DS, including problems with multitasking, cognitive flexibility and working memory.^28–30^ As these are abilities necessary in daily life and in many working environments, this could contribute to the low percentage of participants in full-time, unadjusted employment and the increased need for adjusted work. Future studies should investigate to what extent medical problems such as fatigue^31^ and rheumatic disorders^2^ contribute to the limited number of adults with 22q11.2DS with a regular, full-time job, and other functional outcomes. In contrast to the findings of previous studies in adults with 22q11.2DS, only a small percentage of our sample was married.^8,17^ Females (17.1%) were more often married than males (8.4%). This may have been because of the large numbers of individuals with intellectual disability and low levels of adaptive functioning in our sample. These low levels of adaptive functioning could also explain the large number of participants living with their parents/caregivers or in intellectual disability residential care. Our results indicate that the majority of adults with 22q11.2DS require substantial support in their daily living situation, despite daily living skills being a relative strength of their adaptive functioning.

### Strengths and limitations

An important strength of this study was the large sample size, with all participants recruited from specialised 22q11.2DS referral centres in the Netherlands and Flanders. In addition, structured and objective/standardised assessments (IQ, adaptive functioning, psychiatric) were available for the majority of individuals. Some limitations must be taken into account as well. An important limitation of the present study was its retrospective nature. In addition, assessment of adaptive functioning was not available for all individuals, as this is not part of standard care but is performed when indicated, possibly causing ascertainment bias. However, we recruited participants through several out-patient clinics and scientific research studies, aiming to generate as representative a sample as possible. Unfortunately, we did not have information about the source and reason for referral to the specialised clinics for all participants. Adults with 22q11DS are often referred to specialised clinics because of medical or psychiatric problems. This could have influenced the results of our study. Finally, because of the retrospective nature of the study, we used a dichotomous approach to assess psychopathology instead of a more dimensional approach. Therefore, subclinical symptoms were not taken into account but can in practice have an impact on adaptive abilities and functional outcomes.

### Clinical implications

The results of the current study contribute to our growing knowledge on daily life functioning of adults with 22q11.2DS and may help healthcare providers to counsel adults with 22q11.2DS and their families. Importantly, the results also highlight the need for an adequate and up-to-date diagnostic profile in adults with 22q11.2DS, focusing not only on IQ but also on adaptive functioning, functional outcomes (living situation, job) and psychopathology. A complete profile of skills across different domains will help adults with 22q11.2DS, their caregivers and healthcare professionals to set realistic goals to improve functioning and self-sufficiency and will inform healthcare professionals about suitable interventions. Although not specifically measured in this study, assessment of executive functions could also provide valuable information about how best to increase competencies and find suitable employment in individual cases.^2^ In addition, it is important to take into account medical comorbidities (which are frequently present in this population) to support, guide and advise adults with 22q11.2DS with respect to a realistic living situation and job.^2^ Our results also emphasise the need for targeted and tailored support for adults with 22q11.2DS and for good access to (mental) healthcare services.

A low percentage of adults were being treated in a (long-stay) psychiatric care setting or living in a psychiatric sheltered housing facility at the time of assessment. In regular mental healthcare, higher levels of self-sufficiency and independence are generally expected, which may not match the support needs of adults with 22q11.2DS. An intellectual disability setting, where practitioners typically are better equipped to match these needs, may be a suitable alternative.

### Future directions

The current findings suggest that low levels of adaptive and cognitive functioning are common in adults with 22q11.2DS. In addition, males with 22q11.2DS display lower levels of adaptive functioning and are more likely to have an intellectual disability, psychotic disorder and ASD than females with 22q11.2DS. Moreover, FSIQ and presence of ASD consistently predicted adaptive function across all separated domains of the VABS, highlighting the pervasiveness of these problems and the need for tailored support in these areas. Future longitudinal and multicentre studies including older patients are needed to further investigate cognitive and adaptive trajectories and the interactions with physical and psychiatric comorbidities. Future studies should specifically pay attention to the impact of individual characteristics, such as medical factors, neuropsychological factors (e.g. executive functions), psychiatric comorbidities, family (parenting style, coping styles) and environmental factors (risk and protective factors, adaptive learning environment) as well as therapies and/or interventions on these trajectory patterns. Understanding these diverse developmental outcomes and changes are crucial to providing tailored support and care. Increasing our knowledge of the developmental trajectories in 22q11.2DS will help to identify profiles of clinical need which can guide intervention and treatment decisions, with the ultimate goal of optimising quality of life for all individuals.

## Supporting information

Vingerhoets et al. supplementary material Vingerhoets et al. supplementary material

## Data Availability

The data that support the findings of this study are available from the corresponding author (C.V.) upon reasonable request.

## References

[1] Blagojevic C, Heung T, Theriault M, Tomita-Mitchell A, Chakraborty P, Kernohan K, et al. Estimate of the contemporary live-birth prevalence of recurrent 22q11.2 deletions: a cross-sectional analysis from population-based newborn screening. CMAJ Open 2021; 9(3): E802–9. 10.9778/cmajo.20200294 PMC8373039 34404688

[2] Boot E, Óskarsdóttir S, Loo JCY, Crowley TB, Orchanian-Cheff A, Andrade DM, et al. Updated clinical practice recommendations for managing adults with 22q11.2 deletion syndrome. Genet Med 2023; 25: 100344. 36729052 10.1016/j.gim.2022.11.012

[3] Zinkstok JR, Boot E, Bassett AS, Hiroi N, Butcher NJ, Vingerhoets C, et al. Neurobiological perspective of 22q11.2 deletion syndrome. Lancet Psychiatry 2020; 6(11): 951–60. 10.1016/S2215-0366(19)30076-8 PMC7008533 31395526

[4] Óskarsdóttir S, Boot E, Crowley TB, Loo JCY, Arganbright JM, Armando M, et al. Updated clinical practice recommendations for managing children with 22q11.2 deletion syndrome. Genet Med 2023; 25: 100338. 36729053 10.1016/j.gim.2022.11.006

[5] Vorstman JAS, Breetvelt EJ, Duijff SN, Eliez S, Schneider M, Jalbrzikowski M, et al. Cognitive decline preceding the onset of psychosis in patients with 22q11.2 deletion syndrome. JAMA Psychiatry 2015; 72(4): 377–85. 25715178 10.1001/jamapsychiatry.2014.2671 PMC4383767

[6] Evers LJM, van Amelsvoort TAMJ, Candel MJJM, Boer H, Engelen JJM, Curfs LMG. Psychopathology in adults with 22q11 deletion syndrome and moderate and severe intellectual disability. J Intellect Disabil Res 2014; 58(10): 915–25. 24528781 10.1111/jir.12117

[7] Fiksinski AM, Schneider M, Zinkstok J, Baribeau D, Chawner SJRA, Vorstman JAS. Neurodevelopmental trajectories and psychiatric morbidity: lessons learned from the 22q11.2 deletion syndrome. Curr Psychiatry Rep 2021; 24(3): 13. 10.1007/s11920-021-01225-z PMC7904715 33625600

[8] Butcher NJ, Chow EW, Costain G, Karas D, Ho A, Bassett AS. Functional outcomes of adults with 22q11.2 deletion syndrome. Genet Med 2012; 14(10): 836–43. 22744446 10.1038/gim.2012.66 PMC3465579

[9] van Amelsvoort T, Henry J, Morris R, Owen M, Linszen D, Murphy K, et al. Cognitive deficits associated with schizophrenia in velo-cardio-facial syndrome. Schizophr Res 2004; 70(2): 223–32. 15329299 10.1016/j.schres.2003.10.004

[10] Henry JC, van Amelsvoort T, Morris RG, Owen MJ, Murphy DGM, Murphy KC. An investigation of the neuropsychological profile in adults with velo-cardio-facial syndrome (VCFS). Neuropsychologia 2002; 40(5): 471–8. 11749977 10.1016/s0028-3932(01)00136-1

[11] Leader G, Curtin A, Shprintzen RJ, Whelan S, Coyne R, Mannion A. Adaptive living skills, sleep problems, and mental health disorders in adults with 22q11.21 deletion syndrome. Res Dev Disabil 2023; 136: 104491. 36965410 10.1016/j.ridd.2023.104491

[12] Sparrow S, Balla D, Cicchetti D. Vineland Adaptive Behavior Scales. American Guidance Service, 1984.

[13] Swillen A, McDonald-Mcginn D. Developmental trajectories in 22q11.2 deletion syndrome. Am J Med Genet C Semin Med Genet 2015; 169(2): 172–81. 25989227 10.1002/ajmg.c.31435 PMC5061035

[14] Fiksinski AM, Bearden CE, Bassett AS, Kahn RS, Zinkstok JR, Hooper SR, et al. A normative chart for cognitive development in a genetically selected population. Neuropsychopharmacology 2022; 47(7): 1379–86. 33782512 10.1038/s41386-021-00988-6 PMC9117666

[15] Curtin A, Mannion A, Shprintzen RJ, Whelan S, Coyne R, Leader G. An examination of adaptive behavior and functional outcomes in adults with 22q11.2 deletion syndrome: a parental perspective. Am J Med Genet A 2022; 188(4): 1040–7. 34908218 10.1002/ajmg.a.62604

[16] Mosheva M, Pouillard V, Fishman Y, Dubourg L, Sofrin-Frumer D, Serur Y, et al. Education and employment trajectories from childhood to adulthood in individuals with 22q11.2 deletion syndrome. Eur Child Adolesc Psychiatry 2019; 28(1): 31–42. 29934817 10.1007/s00787-018-1184-2

[17] Mosheva M, Eyal S, Weisman O, Gilad R, Fishman Y, Weinberger R, et al. Higher adaptive functioning and lower rate of psychotic comorbidity in married versus unmarried individuals with 22q11.2 deletion syndrome. Am J Med Genet A 2018; 176(11): 2365–74. 29171699 10.1002/ajmg.a.38555

[18] Velthorst E, Levine SZ, Henquet C, de Haan L, van Os J, Myin-Germeys I, et al. To cut a short test even shorter: reliability and validity of a brief assessment of intellectual ability in schizophrenia – a control–case family study. Cogn Neuropsychiatry 2012; 18: 37–41. 10.1080/13546805.2012.731390 23167265

[19] Calkins ME, Merikangas KR, Moore TM, Burstein M, Behr MA, Satterthwaite TD, et al. The Philadelphia Neurodevelopmental Cohort: constructing a deep phenotyping collaborative. J Child Psychol Psychiatry 2015; 56(12): 1356–69. 25858255 10.1111/jcpp.12416 PMC4598260

[20] Swillen A. The importance of understanding cognitive trajectories: the case of 22q11.2 deletion syndrome. Curr Opin Psychiatry 2016; 29: 133–7. 26779858 10.1097/YCO.0000000000000231 PMC5414032

[21] Cuypers M, Tobi H, Naaldenberg J, Leusink GL. Linking national public services data to estimate the prevalence of intellectual disabilities in The Netherlands: results from an explorative population-based study. Public Health 2021; 195: 83–8. 34062276 10.1016/j.puhe.2021.04.002

[22] McQuaid GA, Pelphrey KA, Bookheimer SY, Dapretto M, Webb SJ, Bernier RA, et al. The gap between IQ and adaptive functioning in autism spectrum disorder: disentangling diagnostic and sex differences. Autism 2021; 25(6): 1565–79. 33715473 10.1177/1362361321995620 PMC8324508

[23] Schneider M, Debbané M, Bassett AS, Chow EWC, Fung WLA, van den Bree M, et al. Psychiatric disorders from childhood to adulthood in 22q11.2 deletion syndrome: results from the international consortium on brain and behavior in 22q11.2 deletion syndrome. Am J Psychiatry 2014; 171(6): 627–39. 24577245 10.1176/appi.ajp.2013.13070864 PMC4285461

[24] Tang SX, Yi JJ, Calkins ME, Whinna DA, Kohler CG, Souders MC, et al. Psychiatric disorders in 22q11.2 deletion syndrome are prevalent but undertreated. Psychol Med 2014; 44(6): 1267–77. 24016317 10.1017/S0033291713001669 PMC4461220

[25] Hendriksen JGM, Peijnenborgh JCAW, Aldenkamp AP, Vles JSH. Diagnostic overshadowing in a population of children with neurological disabilities: a cross sectional descriptive study on acquired ADHD. Eur J Paediatr Neurol 2015; 19(5): 521–4. 25976065 10.1016/j.ejpn.2015.04.004

[26] Aleman A, Kahn R, Selten JP. Sex differences in the risk of schizophrenia evidence from meta-analysis. Arch Gen Psychiatry 2003; 60(6): 565–71. 12796219 10.1001/archpsyc.60.6.565

[27] Maenner MJ, Shaw KA, Bakian AV, Bilder DA, Durkin MS, Esler A, et al. Prevalence and characteristics of autism spectrum disorder among children aged 8 years – Autism and Developmental Disabilities Monitoring Network, 11 sites, United States, 2018. MMWR Surveill Summ 2021; 70(11); 1–16. 10.15585/mmwr.ss7011a1 PMC8639024 34855725

[28] Maeder J, Zuber S, Schneider M, Kliegel M, Eliez S. Age-related improvements in executive functions and focal attention in 22q11.2 deletion syndrome vary across domain and task. J Int Neuropsychol Soc 2022; 28(4): 337–50. 33952381 10.1017/S135561772100059X

[29] Schneider M, Eliez S, Birr J, Menghetti S, Debbane M, van der Linden M. Multitasking abilities in adolescents with 22q11.2 deletion syndrome: results from an experimental ecological paradigm. Am J Intellect Dev Disabil 2016; 121(2): 151–64. 26914469 10.1352/1944-7558-121.2.151

[30] Maeder J, Schneider M, Bostelmann M, Debbané M, Glaser B, Menghetti S, et al. Developmental trajectories of executive functions in 22q11.2 deletion syndrome. J Neurodev Disord 2016; 8: 10. 27018204 10.1186/s11689-016-9141-1 PMC4807556

[31] Vergaelen E, Claes S, Kempke S, Swillen A. High prevalence of fatigue in adults with a 22q11.2 deletion syndrome. Am J Med Genet A 2017; 173(3): 858–67. 28190295 10.1002/ajmg.a.38094

