# Supplementary material for: Cognitive, adaptive and daily life functioning in adults with 22q11.2 deletion syndrome
Source: BJPsych Open. 2024 Nov 11;10(6):e203. doi: 10.1192/bjo.2024.745 (PMC11698185; doi:10.1192/bjo.2024.745)
Supplement: Vingerhoets et al. supplementary material [file S2056472424007452sup001.docx]

**Supplementary material**

**Table 1.** Demographic variables

|  | **Total sample**  **N=250**  **M (SD)** | **Maastricht**  **N=127**  **M (SD)** | **‘s Heeren Loo**  **N=57**  **M (SD)** | **Leuven**  **N=66**  **M (SD)** |
| --- | --- | --- | --- | --- |
| Age functional outcomes  Age last IQ  Age last VABS | 28.66 (11.95)  26.98 (11.15)  30.29 (10.26) | 31.03 (10.51)  30.92 (10.74)  31.13 (10.71) | 32.42 (15.21)  26.83 (12.98)  31.09 (14.84) | 20.83 (6.96)  19.77 (5.57)  26.60 (4.05) |
| **IQ instrument** | **N** |  |  |  |
| WAIS III, shortened | 116 |  |  |  |
| WAIS III, full version | 55 |  |  |  |
| WAIS IV | 32 |  |  |  |
| WISC III | 5 |  |  |  |
| WISC V | 7 |  |  |  |
| WISC, version unknown | 4 |  |  |  |
| Instrument unknown | 27 |  |  |  |
| Not testable | 4 |  |  |  |

*NB: IQ: intelligence quotient; VABS: Vineland Adaptive Behaviour Scale. WAIS: Wechsler Adult Intelligence Scale; WISC: Wechsler Intelligence Scale for Children.*

**Table 2.** Overview of FSIQ and level of ID

| **FSIQ level of ID** | **DSM 5 level of ID** | **N** | **FSIQ range** |
| --- | --- | --- | --- |
| Borderline IQ | Mild | 9 | 71-76 |
| Mild ID | Moderate | 17 | 51-70 |
| Mild ID | Severe | 2 | 60-78 |
| Moderate | Mild | 1 | 46 |

*NB: FSIQ: Full scale intelligence quotient. FSIQ level of ID: level of ID solely based on FSIQ; DSM 5 level of ID: level of ID according to DSM 5.*

**Table 3.** Prevalence of psychopathology

|  | **N** | **%** |
| --- | --- | --- |
| Depression  MDD without psychotic features  Persistent depressive disorder (dysthymia)  Adjustment disorders with depressed mood  Unspecified depressive disorder  Subtype unknown | 47/243  3/47  1/47  1/47  29/47  13/47 | 19.3  6.4  2.1  2.1  61.7  27.7 |
| Bipolar disorder | 2/243 | 0.8 |
| Anxiety disorder  Panic disorder  Agoraphobia  Panic disorder with agoraphobia  Specific phobia  Social anxiety disorder  Generalized anxiety disorder  Unspecified anxiety disorder  Unknown | 38/244  7/38  4/38  2/38  2/38  4/38  14/38  6/38  10/38 | 15.6  15.5  10.5  5.3  5.3  10.5  36.8  15.8  26.3 |
| Psychotic disorder  Schizophrenia  Psychotic disorder due to medical condition  Unspecified psychotic disorder  Unknown | 51/244  15/51  2/51  17/51  17/51 | 20.4  29.4  3.9  33.3  33.3 |
| ADHD  Predominantly inattentive  Combined subtype  Unknown | 10/141  3/10  3/10  2/10 | 7.1  30  30  20 |
| ASD | 47/210 | 22.4 |

*NB: MDD: major depressive disorder; ADHD: attention deficit hyperactivity disorder; ASD: autism spectrum disorder.*

***Sex differences***

**Table 4.** Prevalence of intellectual disability (ID)

|  | **Male** | | **Female** | |  |
| --- | --- | --- | --- | --- | --- |
|  | **N** | **%** | **N** | **%** | **p** |
| No ID | 8/109 | 7.3% | 9/140 | 6.4% | .777 |
| Borderline intelligence* | 31/109 | 28.4% | 59/140 | 41.8% | .026 |
| Mild ID | 50/109 | 45.9% | 58/140 | 41.1% | .483 |
| Moderate ID | 15/109 | 13.8% | 10/140 | 7.1% | .090 |
| Severe/profound ID | 5/109 | 4.6% | 4/140 | 2.8% | .472 |

**Table 5.** Adaptive functioning.

| **Level of adaptive functioning** | **Male** | | **Female** | |  |
| --- | --- | --- | --- | --- | --- |
|  | **N** | **%** | **N** | **%** |  |
| High | 0 | 0.0 | 0 | 0.0 |  |
| Moderately high | 0 | 0.0 | 1 | 1.0 |  |
| Adequate | 8 | 11.9 | 20 | 20.8 |  |
| Moderately low | 9 | 13.4 | 19 | 19.8 |  |
| Low | 50 | 74.6 | 56 | 58.3 |  |
| **Adaptive function scores** | **Male** | | **Female** | |  |
|  | **Standardized score** | **Age equivalent** | **Standardized score** | **Age equivalent** | **p** |
| Communication | 48.24 | 8;5 | 56.48 | 9;7 | .075 |
| Daily living skills* | 60.96 | 9;9 | 74.92 | 12;5 | .008 |
| Socialization | 55.57 | 8;7 | 63.81 | 10;6 | .067 |
| Overall adaptive functioning* | 51.82 |  | 62.30 |  | .014 |

**Table 6.** Prevalence of psychopathology

|  | **Male** | | **Female** | |  |
| --- | --- | --- | --- | --- | --- |
|  | **N** | **%** | **N** | **%** | **p** |
| Psychopathology | 64/109 | 58.7% | 66/141 | 46.8% | .403 |
| Depression | 20/109 | 18.3% | 27/141 | 19.3% | .851 |
| Bipolar disorder | 2/109 | 1.8% | 0/141 | 0.0% | .108 |
| Anxiety disorder | 12/109 | 11% | 26/141 | 18.6% | .100 |
| Psychotic disorder* | 31/109 | 28.4% | 20/141 | 14.3% | .006 |
| ADHD | 6/109 | 5.5% | 4/141 | 2.9% | .291 |
| Autism* | 28/109 | 25.7% | 19/141 | 13.7% | .017 |

**Table 7**. Functional outcomes

|  | **Male** | | **Female** | |
| --- | --- | --- | --- | --- |
|  | **N** | **%** | **N** | **%** |
| **Marital status**  Single (never married)  Married  Long-term relationship  Divorced  Widowed | 89/106  9/106  5/106  2/106  1/106 | 83.2  8.4  5.6  1.9  0.9 | 99/140  24/140  11/140  3/140  3/140 | 70.7  17.1  7.9  2.1  2.1 |
| **Living situation**  With parents/family  With partner/own family  Independent (alone)  Protected environment (alone)  With roommates  ID setting  Psychiatric setting  Other | 44/106  14/106  12/106  4/106  0/106  28/106  4/106  0/106 | 41.5  13.2  11.3  3.8  0.0  26.4  3.8  0.0 | 48/140  38/140  15/140  8/140  2/140  22/140  5/140  2/140 | 34.3  26.4  10.7  5.7  1.4  15.7  3.6  1.4 |
| **Work/daycare**  Sheltered work  Regular job part-time  Regular job full-time  Household  Volunteer work  Day center  School  Unemployed/no daytime activities | 30/106  11/106  14/106  2/106  1/106  32/106  7/106  9/106 | 28.3  10.4  13.2  1.9  0.9  30.2  6.6  8.5 | 28/137  29/137  5/137  6/137  6/137  31/137  16/137  16/137 | 20.4  21.2  3.6  4.4  4.4  22.6  11.7  11.7 |

**Sensitivity analyses**

**Table 8.** Demographic information for subjects ≥ 18 years.

|  | **Total sample**  **N=215**  **M (SD)** |
| --- | --- |
| Sex, male/female | 90/125 |
| Age | 30.7 (11.7) |
| FSIQ | 69.9 (11.5) |
| VIQ | 71.0 (12.4) |
| PIQ | 68.4 (13.8) |
| *De novo*/fam./unknown | 117/19/79 |

*Nb: FSIQ: full scale IQ; VIQ: verbal IQ; PIQ: performance IQ; fam: familial.*

| **Level of intellectual functioning** | **N**  **(total 215)** | | **%** | |
| --- | --- | --- | --- | --- |
| Average intelligence | 12 | | 5.6 | |
| Borderline intelligence | 77 | | 35.8 | |
| Mild ID | 96 | | 44.7 | |
| Moderate ID | 21 | | 9.8 | |
| Severe/profound ID | 9 | | 4.2 | |
| **Level of adaptive functioning** | **N**  **(total 140)^a^** | | **%** | |
| High | 0 | | 0.0 | |
| Moderately high | 1 | | 0.7 | |
| Adequate | 27 | | 19.3 | |
| Moderately low | 28 | | 20.0 | |
| Low | 84 | | 60.0 | |
| **Adaptive functioning**  **domain scores** | **N** | **Standardized score** | | **Age equivalent** |
| Communication | 139 | 57.3 | | 10;12 |
| Daily living skills | 139 | 75.8 | | 12;8 |
| Socialization | 139 | 65.8 | | 10;9 |
| Overall adaptive functioning | 140 | 62.6 | |  |

**Table 9.** Level of function of subjects ≥ 18 years.

*Nb: Average intelligence: FSIQ 85-115; Borderline intelligence: FSIQ 70-84; Mild ID: FSIQ 50-69; Moderate ID: FSIQ 35-49; Severe/profound ID FSIQ <35; Adaptive functioning classifications based on standard scores: High: 131-160; Moderately high: 116-130; Adequate: 85-115; Moderately low: 70-84; Low: 20-69.*

**Table 10.** Predictors of adaptive functioning in subjects ≥ 18 years.

|  | **Overall adaptive functioning** | | **Communication** | | **Daily living skills** | | **Socialization** | |
| --- | --- | --- | --- | --- | --- | --- | --- | --- |
|  | ***B*** | ***P*** | ***B*** | ***P*** | ***B*** | ***P*** | ***B*** | ***P*** |
| Age | 0.56 | .002 | 0.20 | .045 | 0.77 | **<.001** | 0.53 | **.009** |
| Sex | 10.28 | **.007** | 7.99 | .065 | 13.92 | **.003** | 7.76 | .069 |
| FSIQ | 0.94 | **<.001** | 1.10 | **<.001** | 0.88 | **<.001** | 0.67 | **<.001** |
| Depression | 3.28 | .457 | 10.34 | .040 | 0.45 | .931 | 1.15 | .816 |
| Psychosis | 3.68 | .454 | 1.59 | .775 | 2.852 | .627 | 6.12 | .267 |
| Anxiety | -5.69 | .246 | -13.30 | **.018** | 0.58 | .920 | -1.61 | .770 |
| ADHD | 12.58 | .231 | 9.82 | .410 | 10.19 | .416 | 17.85 | .130 |
| ASD | -12.52 | **.016** | -12.08 | **.046** | -13.65 | **.032** | -14.32 | **.017** |

*Nb: ﻿Numbers in bold reflect significant predictors; FSIQ: full scale intelligence quotient; ADHD: attention deficit hyperactivity disorder; ASD: autism spectrum disorder.*
